# Clinical Research Progress of Internal Brace Ligament Augmentation Technique in Knee Ligament Injury Repair and Reconstruction: A Narrative Review

**DOI:** 10.3390/jcm12051999

**Published:** 2023-03-02

**Authors:** Wenhao Lu, Zhenhan Deng, Anko Elijah Essien, Djandan Tadum Arthur Vithran, Michael Opoku, Yusheng Li, Wenfeng Xiao

**Affiliations:** 1 Department of Orthopaedics, Xiangya Hospital, Central South University, Changsha 410008, China; 2 Department of Sports Medicine, The First Affiliated Hospital of Shenzhen University, Shenzhen Second People’s Hospital, Shenzhen 518035, China; 3 National Clinical Research Center for Geriatric Disorders, Xiangya Hospital, Central South University, Changsha 410008, China

**Keywords:** internal brace, internal brace ligament augmentation, knee, ligament repair, ligament reconstruction

## Abstract

Knee ligament injuries are most common in sports injuries. In general, ligament repair or reconstruction is necessary to restore the stability of the knee joint and prevent secondary injuries. Despite advances in ligament repair and reconstruction techniques, a number of patients still experience re-rupture of the graft and suboptimal recovery of motor function. Since Dr. Mackay’s introduction of the internal brace technique, there has been continuous research in recent years using the internal brace ligament augmentation technique for knee ligament repair or reconstruction, particularly in the repair or reconstruction of the anterior cruciate ligament. This technique focuses on increasing the strength of autologous or allograft tendon grafts through the use of braided ultra-high-molecular-weight polyethylene suture tapes to facilitate postoperative rehabilitation and avoid re-rupture or failure. The purpose of this review is to present detailed research progress in the internal brace ligament enhancement technique of knee ligament injury repair as well as the reconstruction from biomechanical and histological research and clinical studies and to comprehensively assess the value of the application of this technique.

## 1. Introduction

Knee ligament injuries are common, predominantly in adolescents, more in males than females, and often occur during traumatic injuries or athletic events such as soccer, basketball, skiing, wrestling, etc. [1,2] The knee ligaments are the primary structures that maintain the stability of the knee joint, providing around 85% of the static resistance to prevent the tibia from moving forward in maintaining the anterior knee stability [3]. Once one or more major ligaments are ruptured, not only is the balance between knee mobility and stability broken, but the receptors of proprioception, which provide information about motion and position, are also disrupted, resulting in knee instability [3,4,5,6]. The anterior cruciate ligament (ACL), posterior cruciate ligament (PCL), medial collateral ligament (MCL), medial patellofemoral ligament (MPFL), and posterior lateral complex (PLC) are key structures in maintaining knee stability [7]. Knee instability caused by knee ligament rupture has been identified as a risk factor for meniscal and cartilage injury, which can significantly increase the risk of osteoarthritis in the patellofemoral and tibiofemoral joints [8,9,10,11].

Studies have shown that knee injuries account for 60% of all sports injuries [12,13]. The ACL is one of the most vulnerable ligaments, and its incidence has been steadily increasing since the last century [1,14,15]. In general, repair or reconstructive surgery after a ligament rupture is necessary to restore stability to the injured knee and prevent further tissue damage [16,17,18,19].

Ligament reconstruction is mainly performed with allograft tendons or autologous tendons such as Bone-Patellar Tendon-Bone, Hamstring-Tendon, quadriceps tendon, etc. [20,21]. There are still some complications after ligament reconstruction, such as anterior knee pain, hamstring weakness at the tendon retrieval site, rotational instability, re-rupture and clinical reconstruction failure after ACL reconstruction, with only 63% to 65% of patients returning to their pre-injury level of motion and at least 10.3% of patients experience graft failure within 10 years [22,23,24,25]. Ligament repair is the first surgical technique applied to ligament rupture. It avoids not only tendon grafting, which reduces complications at the tendon extraction site but also preserves the proprioceptive receptors in the natural ligament for better and faster motor recovery [26,27,28,29]. However, due to its high failure rate [29,30,31,32], most physicians prefer ligament reconstruction.

Mackay and colleagues proposed the surgical technique of internal brace ligament augmentation (IBLA), which involves augmenting ligament repair and reconstruction with a high-strength suture tape that strengthens the ligament and acts as a secondary stabilizer, given the limitations and risks of current treatments for either repair or reconstruction after ligament rupture [33]. The results of a recently published study of patients with acute proximal ACL ruptures treated with ACL repair and internal brace reported that at five years after undergoing surgery, 28 patients (82.4%) showed satisfactory outcomes while six patients had re-rupture (17.6%) and underwent revision surgery [34]. Thirty-seven patients who underwent ACL reconstruction combined with IBLA did not have any graft failure or other significant complications at the 1-year postoperative follow-up [35]. The use of the IBLA technique in the repair or reconstruction of other ligaments of the knee, such as the posterior cruciate ligament, medial collateral ligament, medial patellofemoral ligament, etc., also has advantages over traditional methods [36,37,38]. This study will present the detailed research progress of the IBLA technique in knee ligament injury repair and reconstruction from biomechanical, histological and clinical studies and comprehensively evaluate the application value of this technique.

## 2. What Is an Internal Brace?

A ligament augmentation device (LAD) was proposed and designed by Professor Kennedy in North America in 1980 to protect the graft by load sharing and thus improve the success rate of surgery [39], but the technique has faded from clinicians’ view due to biocompatibility issues that can cause reactive synovitis and effusions in the knee joint, increase the risk of infection, and even delay maturation of the autogenous graft in humans [40]. With the development of medical biomaterials, Mackay and colleagues introduced the concept of IBLA [33], which uses artificially woven ultra-high-molecular-weight (UHMW) polyethylene/polyester suture tapes, such as FiberTape (Arthrex, Naples, FL, USA) and Ethibond (Ethicon, Somerville, NJ, USA) [41], to provide additional strength to the healing tissue, allowing early return to sport and preventing re-injury. Once the concept was introduced, it was quickly applied clinically, and in 2013 Dr. Acevedo’s team applied it to the reconstruction of the Spring Ligament Complex (SLC) of the foot [42]. Subsequently, Lubowitz et al., reported enhanced repair of the medial and posterior medial angles of the knee using the internal brace technique in patients with medial knee laxity [43]. As the internal brace technique continues to mature, its application has been gradually expanded to include repair of the anterior talofibular ligament and deltoid ligament in the ankle, anterior and posterior cruciate ligaments, medial and lateral collateral ligaments and medial patellar ligaments in the knee, and ulnar collateral ligaments in the elbow joint [33,42,43,44,45,46,47,48,49,50]. Smith et al. in 2016 detailed IBLA incorporating allogeneic tendons for ACL reconstruction [51], which incorporates FiberTape with collagen coating from Arthrex into allogeneic extremity tendon grafts to increase the strength of the ACL graft. Immediately afterwards, Aboalata et al. reported an arthroscopic technique of internal brace augmentation reconstruction for ACL, in which an independently fixed FiberTape was used intraoperatively to augment the strength of the autologous hamstring tendon graft [52]. In conclusion, the internal brace technique assists in ligament repair and reconstruction by increasing the strength of the graft or its own ligament using artificial high-strength wire tapes, which not only protects the graft or own autologous ligament, but also facilitates the healing of the graft in the early postoperative revascularization phase to avoid re-rupture or failure.

## 3. Knee Ligament Repair or Reconstruction with Internal Brace

### 3.1. ACL Primary Repair or Reconstruction with IBLA

Anterior cruciate ligament (ACL) tears are one of the most common sports injuries. ACL tears’ treatment has evolved rapidly with innovations in medical technology and medical materials. 1903 saw the first description of ACL repair, and it was not until the 1970s and 1980s that it became popular [53,54,55,56]. And by the end of the 20th century, ACL reconstruction alternative repair became the mainstream treatment modality [57,58]. Reconstruction is still an accepted and proven surgical technique for the treatment of ACL tears, and is increasingly being used worldwide [59,60,61]. Following ACL reconstruction, the “ligamentization” of the grafted tendon is a lengthy process that proceeds sequentially through a phase of acute inflammation, hematologic reconstruction, recellularization, and tissue remodeling, eventually assuming physical and mechanical properties similar to those of the original grafted tendon [62]. In the early post-reconstruction period, specifically, there is still inflammation in the knee joint; the ligament blood flow has not been completely reconstructed, and the stiffness of the graft is very low and can easily cause secondary injury, so it is recommended to avoid impact and rotational movement of the knee joint for 3–4 months after surgery [63,64]. When the graft grows and assimilates with the bone to form a new ligament, only then can it perform a stable biomechanical function [65]. However, only 63% to 65% of patients who underwent ACL reconstruction after a rupture returned to their pre-injury level of motion, with at least 10.3% of patients experiencing graft failure within 10 years [24]. ACL proximal tears, type I and II tears, in the modified Sherman classification, have better repair healing potential than distal or medial tears [66,67], avoiding the need for grafts during reconstruction and reducing the associated discomfort at the tendon extraction site while also preserve the proprioceptive fibers of the natural ACL [68,69,70], though they have a higher probability of graft failure [26,29,30,31,32].

Recently, with the development of biomedical materials, internal brace technology may change this situation [46,51,71,72].

#### 3.1.1. Biomechanical Studies

The biomechanical properties of internal brace suture-tapes are key factors in clinical application and have now been analyzed by some experts, as shown in Table 1. Animal models such as pigs and bovines are chosen for most biomechanical tests because their bones and tendons have similar morphology and mechanical function compared to adults [73,74,75,76]. Bachmaier et al., used two strands of “FiberTape” to strengthen three (small diameter graft) and four (standard diameter graft) bovine flexor tendons [77], and after 3000 cycles of tensile loading, found that the deformation of the third and fourth strands was reduced by 50% and 26%, respectively, in comparison to the unstrengthened group, with the final breaking load increased by 64% and 40%, respectively. Noonan obtained similar conclusions after replacing the fixation device of the tibial end of the graft from a tabbed plate to a tibial screw based on Bachmaier’s [78].

Samue et al., performed Suture Augmentation (SA) on 7 mm and 9 mm diameter bovine tendon grafts and found that 7 mm diameter grafts began to shift their share to the lineal band when subjected to a 200 N load and could shift 31% (125 N) by the time they reached a peak load of 400 N, while 9 mm diameter grafts underwent a 300 N load before the transfer occurs, only when the 9 mm diameter graft is subjected to a 300 N load, eventually carrying 20% of the peak load (80 N) [79]. Both Matava’s and Smith’s teams used human bone-patellar ligament-bone grafts for ACL reconstruction in the porcine knee [80,81]. After cyclic tensile loading, the elongation of the grafts was reduced by 31% and 18% in the SA group versus the unaugmented group, respectively. When Wicks performed all-inside ACL reconstruction of the porcine knee using bovine extensor tendon soft tissue allografts under the same conditions, the average cyclic displacement of the graft with internal brace enhancement was 33% less, and the average ultimate and yield loads were 22% and 25% higher, respectively [82].

In a study of rabbit ACL reconstruction using single-strand FiberTape-assisted autologous semitendinosus tendon grafts, the ultimate load and stiffness of the grafts were found to be 1.7 and 1.4 times higher, respectively, at 8 weeks postoperatively than in the tendon reconstruction group alone [83]. The addition of high-strength sutures to the ACL reconstruction technique resulted in minimal changes in baseline biomechanical properties while significantly improving ultimate load, yield load, and cyclic displacement in a weakened graft model with 80% graft resection [71].

Biomechanical studies in these animals have shown one thing: the graft reinforced with a separate internal brace suture tape results in significantly lower elongation, higher ultimate failure load and stiffness of the graft, and no stress shielding of the bone. Then the risk of graft tear after reconstruction will be reduced clinically, especially in small-diameter soft tissue grafts. In a previous study of ACL reconstruction using polydioxanone (PDS) braided tapes to assist autologous patellar tendon reconstruction in goats, micromotion and over-tensioning of the graft due to early postoperative stress masking and subsequent failure of the internal decompression line resulted in delayed fiber bundle remodeling, delayed tendon-bone interface healing, and rapid loss of biomechanical strength [84]. Hence, the suture tape enhancement may fail due to the material of the suture tape. Bachmaier et al., also used an internal brace to enhance the repaired porcine ACL and found that the additional strength of the internal brace reduced the peak load on the repaired structure of the ACL as well as limiting gap formation to less than 3 mm at loads up to 350 N [85]. In a human cadaveric study [86], suture tape augmented ACL reconstruction similarly achieved a full range of motion of the knee joint, with an improved graft stiffness and increased failure load. Thus, an internal brace plays a crucial role in enhancing the stabilization potential of ACL repair under the load that occurs during normal daily activities.

#### 3.1.2. Histological Studies

Good structural remodeling of the graft after ACL reconstruction is the basis for establishing biomechanical strength. Therefore, the impact of the internal brace technique on the “ligamentization” of the graft and the healing of the tendon-bone interface is also crucial to its value. Cook J.L. performed ACL reconstruction in dogs using a 2-strand FiberTape augmented allograft quadriceps tendon under arthroscopy and found ligament remodeling of the grafted tendon at 6 months postoperatively, in addition to which knee stability and function in dogs were not significantly different from normal ACL controls [87]. On this basis, the allogeneic quadriceps tendon enhanced by an internal brace combined with platelet-rich plasma (PRP) allows for faster and stronger tendon-bone healing and graft “ligamentization”, with both techniques synergistically protecting the graft from early failure and OA progression [88]. The biocompatibility of the internal brace suture tape is also a prerequisite for its clinical applicability. Soreide has validated that FiberTape improves the biomechanical properties of intra-articular ligament reconstruction in a rabbit model at 8 weeks [83]. During this period, FiberTape did not adversely affect bone tunnel healing or cause a long-term elevation in inflammation. Smith implanted intact and broken polyethylene suture tapes (FiberTape) into the knee cavity of dogs separately and found no serious complications such as inflammatory/immune reactions, bone erosion, or premature osteoarthritis at six months postoperatively [89], which is consistent with the findings of Cook J.L. [87]. Thus, FiberTape not only showed good biocompatibility in ACL reconstruction in animals without causing severe inflammatory and immune reactions but also in ligament remodeling by ligamentization of tendon grafts and healing of the fascial interface [87,88,89], which is the key to the value of its application.

#### 3.1.3. Clinical Studies

**Figure 1:**
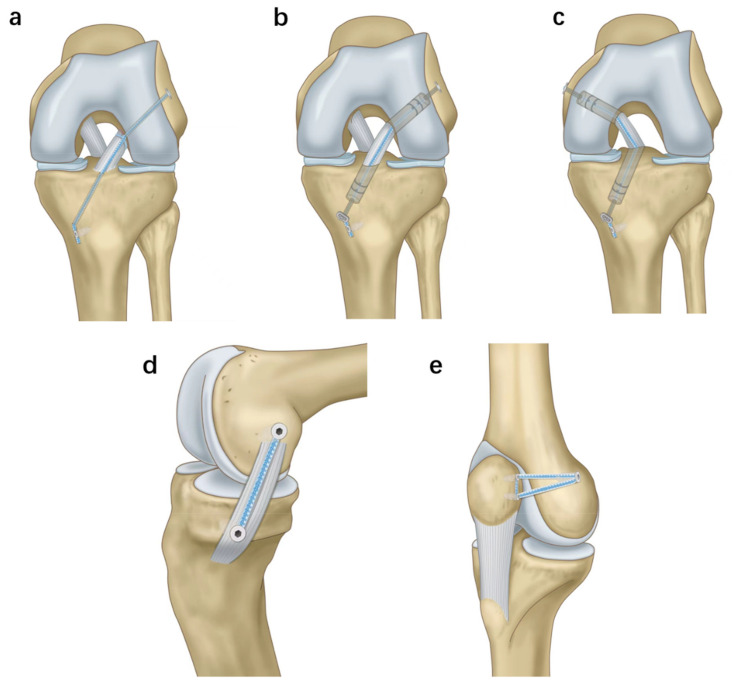
Surgical model of Internal Brace technique in knee ligament injuries. (**a**) Model of ACL repair combined with Internal Brace technology. (**b**) Model of ACL Reconstruction combined with Internal Brace technology. (**c**) Model of PCL Reconstruction combined with Internal Brace technology. (**d**) Model of MCL repair using Internal Brace technology. (**e**) Model of MPFL reconstruction using Internal Brace technique alone.

There have been several clinical studies of ACL repair and reconstruction based on the internal brace technique, as shown in Table 2, all using high-strength suture tapes for arthroscopic ACL repair or reconstruction. As shown in Figure 1a, the ACL repair incorporates internal brace technology, an approach that allows the ACL to heal, bypassing the forces on the supporting FiberTape, thus providing an elegant way to restore physiological function to the knee joint. Victoria et al., performed IBLA repair surgery for ACL on a professional soccer player two weeks after the injury, and the patient was able to play sports without restrictions for six months after surgery and continued to play at the same competitive level for the next 18 months of follow up [90]. Existing studies suggest that augmented ACL repair using suture tapes may increase the cumulative risk of re-rupture with increasing postoperative recovery time, good functional recovery and only a 3% probability of re-rupture at 1 year postoperatively [91], adding a probability of re-rupture ranging from 4.8–14.8% at 2 years postoperatively [92,93,94,95,96]. Hopper et al.’s prospective follow-up study of patients with acute proximal ruptures undergoing ACL repair for at least five years [34] found that 28 patients (82.4%) underwent suture tape-enhanced initial repair of proximal ACL tears showing satisfactory results, while six patients underwent revision surgery when re-rupture occurred (17.6%). No synovitis, erosion, or cyst formation was found in these six patients on further MRI imaging or revision surgery, whereas Tulloch found chronic hypertrophic synovitis during a second arthroscopic procedure using LRAS to reconstruct post-ACL instability [97]. This somewhat reflects the difference between internal bracing techniques and conventional synthetic grafts, and further follow-up is needed to see if synovitis, erosion, and other similar phenomena occur with internal braces older than five years or even 10 years. The results of these studies show a significant improvement compared to the traditional approach to ACL repair, thus suggesting that the late efficacy of the internal brace technique for ACL repair is even comparable to that of standard ACL reconstruction.

ACL reconstruction is now largely performed arthroscopically. The grafts used include homogeneous allografts and autologous tendons such as hamstring tendon, quadriceps tendon, and bone-patellar tendon-bone [20,51,52,98]. The healing process after ACL reconstruction with allografts is longer than with autografts, making the new ligaments less able to withstand the tension caused by knee motion. Patients who receive allografts not only have more than three times the failure rate than those who receive autografts [99] but also have a less subjective patient evaluation with less knee stability than those who receive autologous tendon grafts [100]. Internal brace technology fills this gap by protecting and enhancing homografts [51]. A high-strength suture tape is individually fixed to the femoral and tibial ends to support the protection of the ACL graft in isolation with independent tension. Such a load-sharing device allows the graft to adapt to different levels of strain during the early stages of return to motion until the graft reaches a critical level of strain [52], at which point the reinforced tape can continue to assume greater strain to protect autografts or homografts from irreversible prolongation during the maturation and remodeling phases of healing, as shown in Figure 1b [79]. Bodendorfer et al.’s retrospective analysis of a study of patients who underwent standard ACL reconstruction with autologous popliteal grafts or allografts showed that ACL reconstruction with the combined IBLA technique was associated with improved Patient Reported Outcome Measures, reduced pain, higher percentages, and earlier return to pre-injury-activity-levels [101,102]. A recent study followed 37 patients who underwent ACL reconstruction combined with IBLA for one year postoperatively and found no graft failure or other significant complications to develop [35].

The utilization of the internal brace technique in ACL repair or reconstruction surgery yields manifest benefits, but its efficacy is not absolute. A recent comprehensive analysis of nine studies, spanning from 2014 to 2022, focusing on ACL proximal tears augmented with internal brace repair, uncovered a mean failure rate of 10.4% at an average follow-up period of 2.7 years [103]. At least two years after surgery, there was no significant difference in the need for revision due to re-injury or surgical failure between internal brace enhanced reconstruction and standard ACL reconstruction [101,102]. In Bodendorfer et al.’s study cohort, 6.7% (2/30) of patients in the SA group and standard ACL reconstruction underwent repair due to re-injury [101], which is similar to the results of Parkes’ team’s study cohort, in which 3% (1/36) of patients in the SA group and 6% (4/27) of patients in standard ACL reconstruction experienced graft failure [102]. The proportion requiring reoperation for graft failure, meniscus, arthrofibrosis, and infection in the IB group was consistent with the standard ACL reconstruction group, 14% in Parkes’ study and 13.3% in Bodendorfer’s study [101,102]. The matched cohort studies of Bodendorfer and Parkes were consistent in terms of patient age and knee status prior to surgery and were not statistically different. Notably, the study by Gagliardi et al., manifested the highest failure rate, with 9 of 22 patients experiencing failure, in contrast to the standard ACL reconstruction group, in which six of 157 patients experienced failure [104]. This outcome may be attributed to the fact that the internal brace-enhanced repair group had a lower mean age and percentage of patients with skeletal maturity (13.9 years, 32%) as compared to the standard ACL reconstruction group (15.7 years, 58%). Prior research has indicated that younger patients are more susceptible to recurrent ACL injury, thereby placing them at an augmented risk [105]. Furthermore, activity level and knee laxity are significant factors that intensify the likelihood of surgical failure, with patients who necessitate a high degree of mobility and exhibit pronounced pivotal instability in the knee exhibiting a heightened vulnerability.

**Table 1:** Biomechanical Studies of ACL Primary Repair or reconstruction with IBLA.

Author	Year	Journal	Model	Methods	Outcomes							
					Stiffness (N/mm)		Total Elongation (mm)		Ultimate Failure Load (N)		Yield Load (N)	
					With SA	Without SA	With SA	Without SA	With SA	Without SA	With SA	Without SA
Bachmaier S. et al. [79]	2022	Arthroscopy	Bovine tendon grafts, 7-mm and 9-mm diameter, with and without SA were tested using suspensory fixation. The independent SA was looped over a femoral button and knotted on a tibial button.	Preconditioned constructs were incrementally increased loaded (100 N/1000 cycles) from 100 N to 400 N for 4000 cycles (0.75 Hz) with final pull to failure (50 mm/min).	195.9 ± 9.8 (7 mm, 400 N); 208.9 ± 13.7 (9 mm, 400 N)	113.4 ± 9.3 (7 mm, 400 N); 151.9 ± 13.8 (9 mm, 400 N)	1.90 ± 0.27 (7 mm); 1.50 ± 0.33 (9 mm)	4.77 ± 1.08 (7 mm); 3.57 ± 0.54 (9 mm)	1435 ± 228 (7 mm); 1806 ± 157 (9 mm)	835 ± 92 (7 mm); 1044 ± 49 (9 mm)	NR	NR
Wicks D. E et al. [82]	2022	Orthopaedic Journal of Sports Medicine	All-inside ACL reconstruction of the porcine knee using bovine extensor tendon soft tissue allografts, with or without suture tape reinforcement. The suture tape was placed through the tension loop in the femoral fixation construct and independently fixed in the tibia with an interference screw anchor.	Position-controlled cyclic loading, 50–250 N for 250 cycles, and pull-to-failure (20 mm/min).	136 ± 16 (10.4 ± 0.3 mm)	132 ± 18 (10.4 ± 0.3 mm)	3.9 ± 0.7 (10.4 ± 0.3 mm)	5.8 ± 1.5 (10.4 ± 0.3 mm)	921 ± 180 (10.4 ± 0.3 mm)	717 ± 122 (10.4 ± 0.3 mm)	808 ± 201 (10.4 ± 0.3 mm)	602 ± 155 (10.4 ± 0.3 mm)
Torres S. J. et al. [86]	2022	Arthroscopy, Sports Medicine, and Rehabilitation	Bone - patellar tendon - bone graft in cadaveric knee for ACL reconstruction, with and without suture tape augmentation.	Apply an anterior drawer force of 88 N at 0°, 15°, 30°, 60° and 90° of knee flexion and hold for 15 s to measure displacement. The reconstructed knee is locked in 30° flexion, allowing the tibia to shift forward at a rate of 10 mm/min until failure occurs.	20.3 ± 3.9 (10 mm)	23.5 ± 3.3 (10 mm)	NR	NR	170.4 ± 38.1 (10 mm)	141.8 ± 51.2 (10 mm)	NR	NR
Matava M.J. et al. [80]	2021	Arthroscopy	Human BTB graft fixated between porcine knees, with and without suture tape augmentation. Interference screw fixation.	Cyclic loading between 50–250 N was performed for 500 cycles at 1 Hz, and pull-to-failure (20 mm/min).	261 ± 76 (10 mm)	128 ± 28 (10 mm)	5.87 ± 1.43 (10 mm)	7.16 ± 1.42 (10 mm)	744 ± 219 (10 mm)	473 ± 169 (10 mm)	601 ± 124 (10 mm)	503 ± 137 (10 mm)
Lai V. J. et al. [71]	2021	Arthroscopy, Sports Medicine, and Rehabilitation	ACL reconstruction using bovine extensor tendon in a porcine knee joint model: a SSC, performed with a femoral button and tibial interference screw; and a DSC, with a femoral and tibial button.	Sinusoidal cyclic loading of the samples was conducted for 500 cycles, at 50 to 250 N and 1 Hz, followed by a single cycle load to failure at 20 mm/min.	139 ± 28 (SSC); 128 ± 22 (DSC)	146 ± 28 (SSC); 118 ± 14 (DSC)	4.4 ± 0.7 (SSC); 3.8 ± 0.8 (DSC)	5.3 ± 1.1 (SSC); 5.2 ± 1.9 (DSC)	1000 ± 139 (SSC); 850 ± 216 (DSC)	891 ± 116 (SSC); 747 ± 86 (DSC)	820 ± 186 (SSC); 815 ± 212 (DSC)	826 ± 122 (SSC); 680 ± 147 (DSC)
Bachmaier S. et al. [85]	2020	Orthopaedic Journal of Sports Medicine	ACL repair constructs with single–and double–cinch loop cortical button fixation as well as knotless suture anchor fixation were biomechanically tested with the addition of an internal brace.	Peak load levels ranged from 50 N and increased in 100-N increments to 350 N over 4000 cycles, and pull-to-failure (20 mm/min).	NR		4.00 ± 0.25 (IB-Button, 350 N) 3.82 ± 0.33 (IB-Anchor, 350 N)		NR		NR	
Noonan B.C. et al. [78]	2020	Arthroscopy	Tripled smaller-diameter (8 mm) and quadrupled (9 mm) bovine tendon grafts with and without suture tape reinforcement.	Dynamic testing was performed in position and force control at 250 N and 400 N, followed by pull to failure with the mode of failure noted.	272 ± 19 (8 mm, 400 N); 280 ± 32 (9 mm, 400 N)	176 ± 9 (8 mm, 400 N); 200 ± 10 (9 mm, 400 N)	2.01 ± 0.50 (8 mm); 1.98 ± 0.51 (9 mm)	4.54 ± 0.75 (8 mm); 3.25 ± 0.49 (9 mm)	829 ± 100 (8 mm); 939 ± 76 (9 mm)	1074 ± 148 (8 mm); 1125 ± 157 (9 mm)	NR	NR
Smith P.A. et al. [81]	2020	The Journal of Knee Surgery	ACL reconstruction with human bone-patellar tendon-bone allografts in a porcine model: interference screw fixation on femur and tibia (S-S), adjustable-loop device fixation on the femur with tibial interference screw without suture tape (ALD-S), and with internal brace (ALD-S-IB).	Position-controlled cyclic loading, 50–250 N for 250 cycles, and pull-to-failure (20 mm/min).	156 ± 23 (10 mm, ALD-S-IB)	122 ± 28 (10 mm, ALD-S); 104 ± 40 (10 mm, S-S)	2.9 ± 0.8 (10 mm, ALD-S-IB)	4.2 ± 0.9 (10 mm, ALD-S) 4.3 ± 1.1 (10 mm, S-S)	758 ± 128 (10 mm, ALD-S-IB)	628 ± 233 (10 mm, ALD-S); 416 ± 167 (10 mm, S-S)	NR	NR
Soreide, E et al. [83]	2019	The Bone & Joint Journal	New Zealand rabbits underwent bilateral anterior cruciate ligament reconstruction by autograft, FiberTape, or FiberTape-augmented autograft.	A hydraulic material testing machine was used for assessments of biomechanical properties in a load-to-failure test. the displacement rate was set to 20 mm/min and data were collected via linear potentiome- ter at 200 Hz with a customized LabView program.	21.4 (15.1 to 33.7)	15.1 (11.4 to 26.1)	6.6 (6.1 to 10.0)	9.2 (7.7 to 11.7)	46.1 (35.0 to 64.3)	26.4 (16.6 to 47.2)	NR	NR
Bachmaier S. et al. [77]	2018	Arthroscopy	Tibial and femoral ends were tested using suspension fixation for triple “small” diameter and quadruple "standard" bovine tendon grafts with and without suture tape reinforcement.	Position-controlled cyclic loading, 250 N and 400 N force-controlled cyclic loading, and pull-to-failure (50 mm/min).	219.7 ± 10.7 (8 mm, 400 N); 231.2 ± 29.8 (9 mm, 400 N)	129.4 ± 8.9 (8 mm, 400 N); 156.9 ± 10.4 (9 mm, 400 N)	2.44 ± 0.29 (8 mm); 2.39 ± 0.28 (9 mm)	5.91 ± 0.76 (8 mm); 3.91 ± 0.74 (9 mm)	1592 ± 105 (8 mm); 1585 ± 265 (9 mm)	968 ± 103 (8 mm); 1131 ± 89 (9 mm)	NR	NR

ACL: Anterior Cruciate Ligament; SA: Suture Augmentation; NR: not reported; BTB: Bone-Patellar Tendon-Bone; SSC: single suspensory construct; DSC: double suspensory construct; IB: internal brace.

**Table 2:** Clinical Studies of ACL Primary Repair or reconstruction with IBLA.

Author	Year	Journal	Surgery Type	Study Type	NO. of Patients	Follow-Up	Failure	Outcome Measures
Hopper, G. P. et al. [34]	2022	Knee Surgery, Sports Traumatology, Arthroscopy	Repair	Prospective case series	34	5 years	6 (17.6%)	KOOS, VAS-pain, VR-12 and the Marx Activity scale
Duong, T. D. et al. [35]	2022	Asia-Pacific Journal of Sports Medicine, Arthroscopy, Rehabilitation and Technology	Reconstruction	Prospective Case series	37	1 year	0	IKDC score, LS, ROM, Lachman’s test, anterior drawer test, Pivot shift test
Burton, D. A. et al. [93]	2021	Arthroscopy	Repair	Prospective case series	29	2 years	2 (6.9%)	KOOS and SANE scores
Heusdens, C. H. W. et al. [92]	2021	Knee Surgery, Sports Traumatology, Arthroscopy	Repair	Prospective case series	35	2 years	4 (11.4%)	IKDC score, LS, TS, RTW, RTS, MRI
Parkes, C. W. et al. [102]	2021	Arthroscopy	Reconstruction	Retrospective comparative study	36 (ACL reconstruction with IB) + 72 (ACL standard reconstruction)	2 years	1 (3%) + 4 (6%)	IKDC score, LS, TS, VAS, ROM, Lachman’s test, Pivot shift test
Schneider K. N. et al. [91]	2020	Journal of Clinical Medicine	Repair	Retrospective case series	88	1 year	3 (3.4%)	IKDC score, LS, TS, KT-1000 arthrometer
Douoguih, W. A. et al. [94]	2020	Arthroscopy, Sports Medicine, and Rehabilitation	Repair	Retrospective case series	27	2 years	4 (14.8%)	KOOS, VAS-pain, VR-12 and the Marx Activity scale, and Single Assessment Numeric Evaluation data
Heusdens, C. H. W. et al. [96]	2019	Knee Surgery, Sports Traumatology, Arthroscopy	Repair	Prospective case series	42	2 years	2 (4.8%)	KOOS, VAS-pain, VR-12 and the Marx Activity scale
Jonkergouw, A. et al. [95]	2019	Knee Surgery, Sports Traumatology, Arthroscopy	Repair	Retrospective cohort study	29 (ACL repair) + 27 (ACL repair with SA)	2 years	4 (13.8) +2 (7.4%)	IKDC score, LS, TS, SANE, modified Cincinnati Score
Gagliardi, A. G. et al. [104]	2019	The American Journal of Sports Medicine	Reconstruction and repair	Retrospective cohort study	157 (standard ACLR) + 22 (ACL repair with SA)	2.7 years/ 3.2 years	6 (4.7%) + 9 (44.8%)	IKDC score, LS, ROM, KT-1000 arthrometer, MRI
Bodendorfer, B. M. et al. [101]	2019	Arthroscopy	Reconstruction	Retrospective cohort study	30 (standard ACLR with SA) + 30 (standard ACLR)	2 years	4 (13.3%) + 4 (13.3%)	IKDC score, KOOS, WOMAC, SANE, activity levels
Kumar S. et al. [106]	2019	International Journal of Orthopaedics Sciences	Reconstruction	Prospective cohort study	25 (standard ACLR with SA) + 25 (standard ACLR)	6-months	0	Anterior Drawer test, Lachman test, Pivot shift test, LS
McIntyre V. et al. [90]	2019	Surgical Technology International	Repair	Retrospective case report	1	18-months	/	/

ACLR, Anterior cruciate ligament reconstruction; SA, Suture Augmentation; KOOS, Knee Injury and Osteoarthritis Outcome Score; VAS-pain, Visual Analogue Pain Scale; VR-12, Veterans RAND 12 Item Health Survey; IKDC score, International Knee Documentation Committee score; LS, Lysholm score; ROM, range of motion; SANE, single assessment numerical evaluation; TS Tegner score; RTW, together with return to work; RTS, return to sport; MRI, Magnetic Resonance Imaging; SANE, Single Assessment Numeric Evaluation; WOMAC, Western Ontario and McMaster Universities Osteoarthritis Index.

### 3.2. PCL Internal Brace Repair or Reconstruction

Rupture of the PCL, the strongest ligament in the knee, is rare and usually combined with other ligament or meniscal injuries [107]. Historically, primary PCL repair has been the preferred option; however, PCL reconstruction is currently the widest-used treatment modality. Hopper detailed the use of the internal brace technique for augmented repair of the PCL and prospectively followed 17 patients who underwent suture tape augmented PCL repair for at least two years [108,109], showing greater improvement in pain, motion, and quality of life with a superior success rate compared to conventional PCL reconstruction, which is in agreement with the findings reported by Trasolini [110]. Isolated PCL reconstruction using conventional tendons significantly improves functional knee outcomes and improves knee laxity but results in lower rates of return to pre-injury levels of motion [111]. The PCL reconstruction combined with the internal brace technique is similar to the ACL, as in Figure 1c, with FiberTape sutures connected by a GraftLink button on the femoral side and an anchor nail on the tibia. Levy performed PCL reconstruction using porcine and quadruple bovine tendons, both fixed with a suspension adjustable ring device on the femoral side and screws or a suspension adjustable ring device on the tibial side. The addition of separate suture tapes to the PCL reconstruction was found to reduce total elongation and increase final strength regardless of the fixation method used [112].In fresh human cadavers, it was found that PCL reconstructions enhanced with the internal brace technique showed significantly less tibial translation under cyclic posterior drawer loading of 45 N, 90 N, and 134 N compared to PCL reconstructions alone but did not limit posterior translation of the tibial plateau more than intact physiologic state PCL [113]. The internal brace technique was used as a treatment for acute PCL ruptures of the knee in a single-center study that included 14 cases and ultimately showed good clinical outcomes and MRI imaging reports [37]. These confirmed that IBLA in the clinical setting can reduce knee laxity and provide better joint stability after PCL reconstruction.

### 3.3. MCL Internal Brace Repair

Medial knee injuries have a high probability of occurrence, and most medial knee injuries do not require surgical treatment, except for grade III injuries or combined multiple ligament injuries that may require surgery to maintain knee stability. The anatomical structures involved in maintaining the stability of the posterior medial knee include the MCL, posterior oblique ligament (POL), and the semimembranous tendon. When injuries to these ligaments cause medial laxity of the knee, surgical repair of the posterior medial knee is required to restore stability to the posterior medial corner of the knee [114,115,116]. Particularly, in the case of acute combined posterior medial and ACL injuries with valgus and rotational instability of the knee, the anchors of the femoral MCL and POL footprint areas are loaded with high-strength sutures, thereby enhancing the repair of the superficial medial collateral ligament (sMCL) and POL ligaments and restoring near-instant valgus and external rotation laxity [43,117,118]. Van der List, J.P provided a detailed explanation of a surgery method for acutely repairing the primary MCL using an internal brace through two small incisions, as depicted in Figure 1d, to prevent muscle atrophy and knee valgus instability for early and rapid rehabilitation [38]. This approach is also applicable to acute distal MCL tears [119,120].

The sMCL is the primary constraint for knee valgus 0–90°, whereas the deep medial collateral ligament (dMCL) is the greatest medial constraint for tibial abduction rotation [121]. Thus, after combined ACL and MCL injury, anteromedial rotational instability (AMRI) may persist if sMCL and dMCL healing is inadequate and cannot be restored by isolated single-beam sMCL reconstruction, while MCL deficiency will also increase the risk of ACL reconstruction failure. A dMCL construct oriented anterodistally across the medial joint line, along with an sMCL graft, can restore native knee external rotation laxity [122,123]. Combined reconstruction of the flat sMCL and anteromedial can restore the closest to intact knee kinematics [122]. Both anatomic sMCL-enhanced repair using the internal brace technique as well as anatomic sMCL reconstruction improved knee kinematics with no significant differences in the biomechanical analysis [124], as recently confirmed by a prospective randomized controlled trial published by LaPrade et al. [125]. Fifty-four patients were randomly assigned to two groups, anatomic sMCL reconstruction using autografts and sMCL augmentation repair, and there were no significant differences in patient-reported outcomes between the two groups at one year postoperatively and on lower X-ray valgus stress radiographs. IBLA repair would replace tendon reconstruction techniques, thus clinically reducing failure rates and residual laxity after MCL repair, as well as shorter fixation times with faster return to activity.

### 3.4. MPFL Internal Brace Repair or Reconstruction

Over the past two decades, MPFL reconstruction using allograft tendons or autografts has become the treatment of choice for recurrent patellar dislocation/hemislocation [126]. In a study with up to 10 years of follow-up, good mid-term clinical outcomes were noted for MPFL reconstruction with 97% patient satisfaction. Biomechanical studies have found that synthetic high-strength suture tapes (FiberTape, Arthrex) not only provide higher initial strength than popliteal tendons while preserving the autologous tendon and reducing donor-area complications but are also less prone to relaxation, adjustable in tension, and free of rejection [2]. This technique involves anchoring the central portion of the FiberTap to the medial edge of the patella with two 3.5 mm knotless anchors. Then, with the knee in 60–90 degrees of flexion and the patella maintained in the center of the patellar groove, the ends of the FiberTap are secured to the femoral side with one 4.75 mm knotless anchor nail (Figure 1e) [2,36,127,128,129,130] The double tunnel reconstruction used on the patellar side reduces the risk of patellar fracture due to the smaller diameter of the tunnel, and the double tunnel technique is more consistent with the biomechanics of the patellofemoral joint, reducing the impact on the contact pressure of the patellofemoral joint can better correct patellar tilt and effectively prevent patellar re-dislocation [131]. The suture band augmentation of the MPFL has similar initial patellofemoral contact pressure and joint kinematics compared to MPFL reconstruction using allograft tendons [130]. The MPFL repair technique with suture tape augmentation strengthens the ligament and acts as a secondary stabilizer, protecting the ligament during the healing phase while allowing early movement to promote natural healing while ensuring that the suture tape is not overly constricted [127]. Hopper G.P. found no complications or surgical failures in 18 patients who underwent augmented repair of the MPFL using suture tapes at 5-year postoperative follow-up, with the exception of one missed visit, and most patients reported satisfaction with pain relief and return to physical activity [128]. Lee P.Y.F. prospectively compared the clinical outcomes of MPFL reconstruction using thin femoral tendon autografts and synthetic high-strength suture tapes (FiberTape) [129], with no significant differences in knee function scores between the two groups during the one-, two-, and four-year postoperative follow-up, and no patients were seen to have patellar instability or recurrent dislocation. Xu. et al., found one patient with a secondary lateral patellar dislocation at six months postoperatively at a one-year follow-up of patients who underwent FiberTape reconstruction of the MPFL alone, with a surgical success rate of 94.1% (16/17) [36]. One of the remaining 16 patients had loosening of the medial patellar support band due to loosening of the lateral femoral knotless anchor nail at the last follow-up, but no significant pain during knee flexion and mild activity. Whether fatigue wears with the internal brace leads to failure of the long-term effect, but further clinical studies are needed to determine the long-term effect. Therefore, internal brace replacement of the tendon for MPFL reconstruction is feasible with respect to the current findings.

### 3.5. Anterolateral Ligament (ALL) Internal Brace

ALL is the lateral ligament of the human knee, starting at the lateral epicondyle of the femur and ending at the anterolateral aspect of the proximal tibia [132], mainly to maintain the stability of the knee joint in internal rotation [133,134,135]. Recent surgical and imaging studies have shown that the incidence of the anterolateral ligament (ALL) injuries associated with ACL tears is approximately 90% [136]. In Laboudie’s follow-up observation of 203 patients who underwent initial ACL reconstruction with either a four-strand popliteal graft or a four-strand popliteal graft combined with ALL reconstruction [137], the graft re-rupture rate in the group receiving the four-strand popliteal graft was 11.9%, and 9.9% of patients underwent a second meniscus surgery, whereas the graft in the group receiving the four-strand popliteal graft combined with ALL reconstruction had a rupture rate of 5.8% and only 1.9% of patients underwent a second meniscus surgery. Therefore, surgical repair of ALL combined with ACL reconstruction is an option to achieve anatomic reconstruction in ACL ruptures, while increasing the strength of ALL through an internal brace provides load-sharing and protective properties for its repair and reconstruction. ALL repairs with an internal brace will improve the rotational stability of the knee and accelerate postoperative recovery after ACL reconstruction [138,139,140]. Hopper. found that the combination of ACL repair and internal brace augmentation of ALL demonstrated good patient-reported outcomes in 94.7% of patients [141], then additional ALL surgery in patients at high risk for ACL rupture may better secure rotational stability of the knee.

## 4. Summary and Prospects

Ligament repair or reconstruction combined with IBLA is an emerging surgical technique for the treatment of ligament rupture. IBLA plays a positive role in assisting autologous or allogeneic tendons in ligament repair or reconstruction of the knee joint, mainly in the following aspects: (i) Reduced elongation of the graft under cyclic loading, increased tolerable limit of increased load, enhancing the initial biomechanical properties of the graft; (ii) Good biocompatibility without causing severe inflammatory and immune reactions; (iii) Early postoperative protection of the graft, supporting postoperative ligamentization and direct tendon-bone interface healing to avoid failure due to graft failure; iv. Early and safe rehabilitation; v. Special value for patients with small graft diameter due to thin tendons; vi. Potential advantages for professional athletes and large-weight patients. Despite these advantages of using the endoprosthesis technique, not only is there still a lack of high-quality randomized clinical studies with five or even 10 years of follow-up to support these findings but there is also an increased financial burden on patients if they choose to use endoprosthesis for augmentation reconstruction.

To prevent stress shielding of tendon grafts caused by high-strength wire tapes, independent wire tapes for ligament augmentation are an excellent option. Not only the knee ligament but also the anterolateral ligament of the ankle and the ulnar collateral ligament of the elbow have been reported to have good patient outcomes [49,142,143,144,145,146,147]. In conclusion, IBLA can be used to assist tendon grafts in ligament repair and reconstruction, and its “seatbelt”-like internal decompression device provides stress protection to the graft and has shown promising results for clinical use.

## Data Availability

The datasets used and/or analyzed in the current study are available from the corresponding author upon reasonable request.
